# Supplementary material for: Expression of the Cyanobacterial FoF1 ATP Synthase Regulator AtpΘ Depends on Small DNA-Binding Proteins and Differential mRNA Stability
Source: Microbiol Spectr. 2022 Apr 21;10(3):e02562-21. doi: 10.1128/spectrum.02562-21 (PMC9241938; doi:10.1128/spectrum.02562-21)
Supplement: SUPPLEMENTAL FILE 1 — Supplemental material. Download spectrum.02562-21-s001.pdf, PDF file, 0.5 MB [file spectrum.02562-21-s001.pdf]

Expression of the cyanobacterial F_oF_1 ATP synthase regulator AtpΘ depends on small DNA-binding proteins and differential mRNA stability

Kuo Song[1,#], Martin Hagemann[2], Jens Georg[1], Sandra Maaß[3], Dörte Becher[3], and Wolfgang R. Hess[1*]

[1]Genetics and Experimental Bioinformatics, Faculty of Biology, University of Freiburg, D-79104 Freiburg, Germany;

[2]Plant Physiology Department, Institute of Biological Sciences, University of Rostock, D-18059 Rostock, Germany;

[3]Department of Microbial Proteomics, Institute of Microbiology, Center for Functional Genomics of Microbes, University of Greifswald, D-17489 Greifswald, Germany.

[#]Current address: Institute of Biochemistry and Molecular Biology, ZBMZ, Faculty of Medicine, University of Freiburg, D-79104 Freiburg, Germany

SUPPLEMENTAL MATERIAL

Supplementary figures

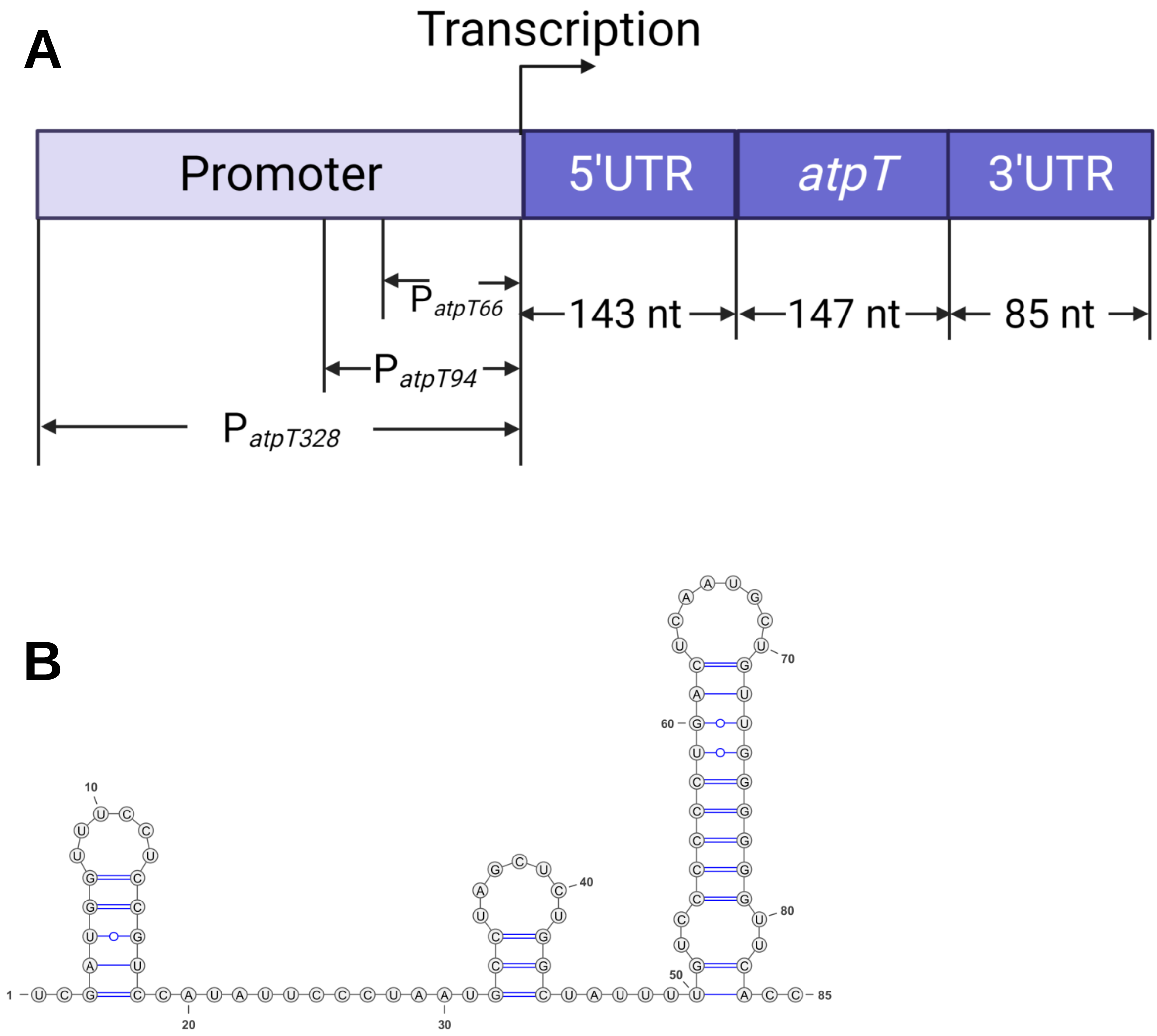

FIG S1 Structure of the *atpT* gene and transcript in *Synechocystis* 6803. (A) The *atpT* transcript consists of a 143 nt 5'UTR, 147 nt coding sequence, and 85 nt 3'UTR. Three *atpT* promoter fragments encompassing 66 nt, 94 nt or 328 nt upstream of the TSS were investigated, called P_{atpT66}, P_{atpT94} and $P_{atpT328}$, as indicated. (B) Predicted RNA secondary structure of the 3'UTR.

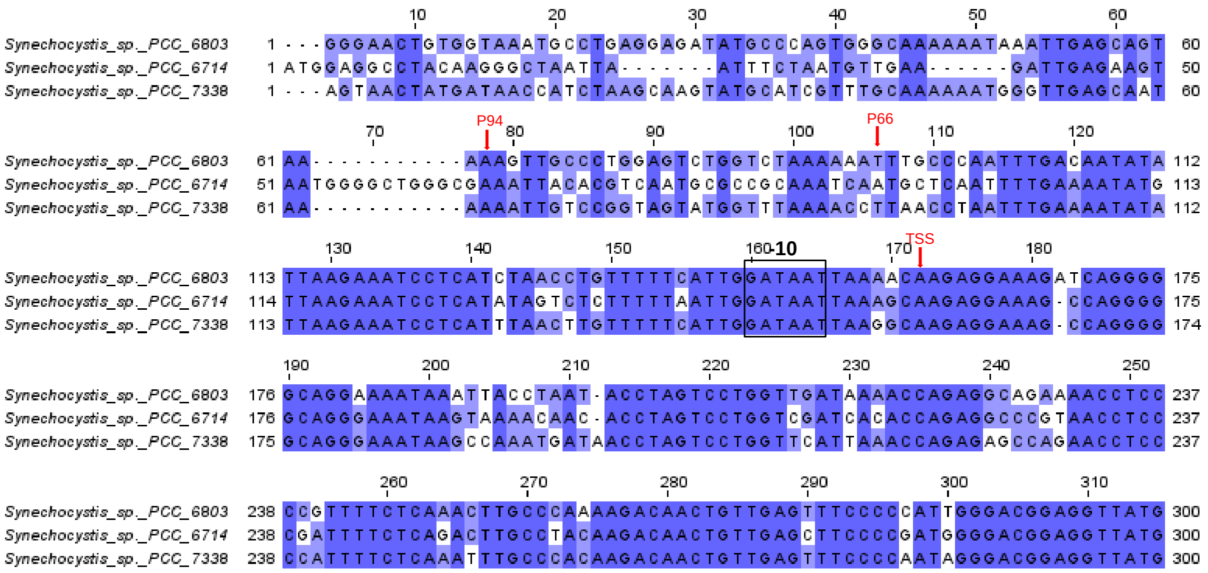

FIG S2 Alignment of the promoter and 5'UTR regions of *atpT* homologs in three *Synechocystis* strains. Dark blue shading indicates identical residues in all three compared strains, and light blue shading indicates identity in the sequences of any two strains. Red arrows mark the TSSs identified previously for *Synechocystis* 6803 and *Synechocystis* 6714 (1, 2). The positions -66 and -94 relative to the TSS in *Synechocystis* 6803 are indicated. These fragments were chosen to define fragments for experimental analyses (compare Fig. S1a). The -10 element according to the mapped TSSs in strains 6803 and 6714 is boxed. For the alignment, 300 nt upstream of the respective *atpT* start codon was selected using MUSCLE (3). Notably, the coding sequence in all three strains starts with two consecutive ATG codons. Here, we considered the second codon as the authentic start codon, while the first was considered the last codon of the 5'UTR. The sequence data were derived from GenBank files NC_000911.1 (*Synechocystis* 6803), CP007542.1 (*Synechocystis* 6714), and CP054306.1 (*Synechocystis* 7338).

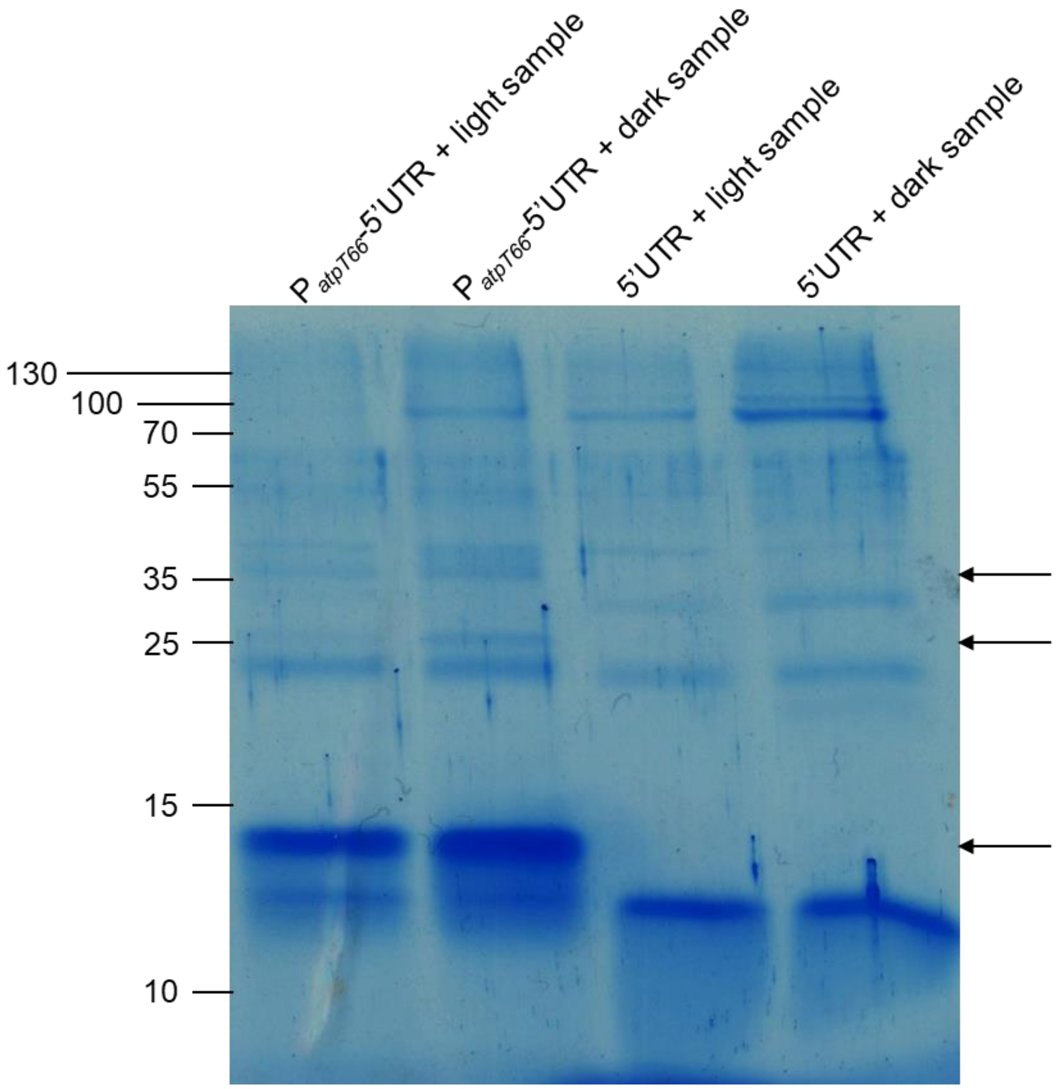

FIG S3 Tricine SDS–PAGE gel showing the sample pulled down using different biotinylated DNA fragments. Two DNA fragments containing the 66 nt-long promoter variant and the 5'UTR (P_{atpT66}-5'UTR) or the *atpT* 5'-UTR were used. These two fragments were incubated with total protein samples isolated from wild type *Synechocystis* 6803 cultured under continuous light or after 12 h in the dark. The arrows indicate bands of significantly enriched proteins interacting with the P_{atpT66}-5'UTR or the *atpT* 5'-UTR DNA fragment. The same samples were subsequently subjected to mass spectrometry analysis. PageRuler™ Prestained Protein Ladder (Thermo Scientific, 10 to 180 kDa) was used as a molecular mass marker.

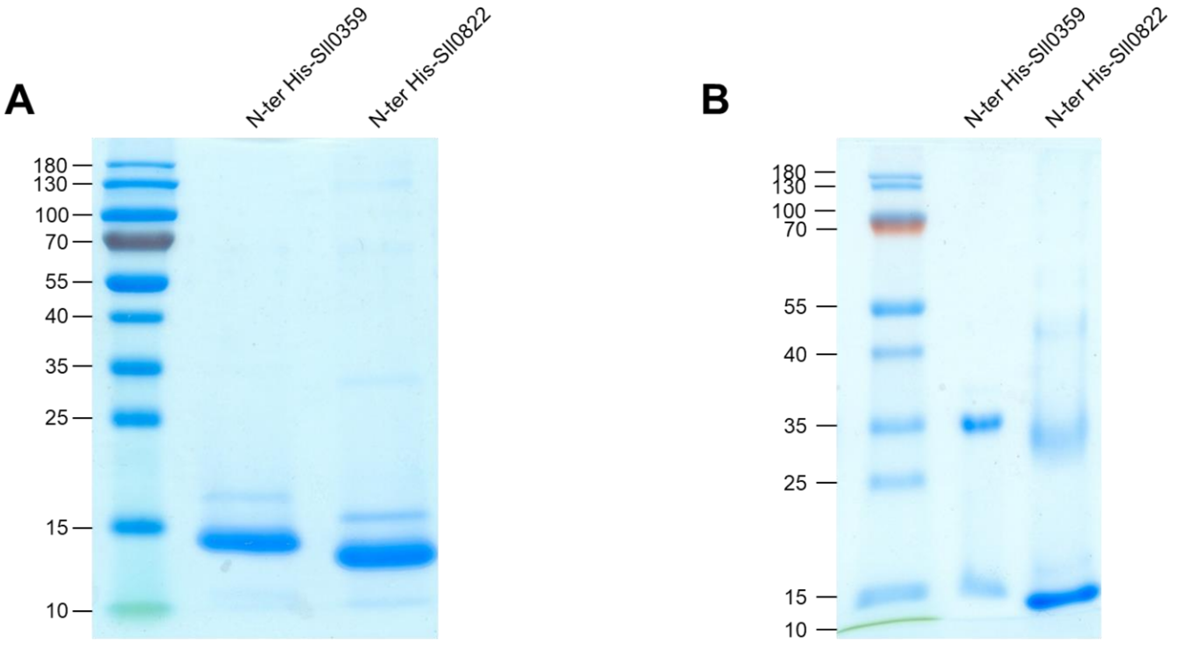

FIG S4 PAGE analysis of the purified Sll0822 and Sll0359 proteins. The recombinant 6*His-tagged Sll0822 and Sll0359 proteins were purified from *E. coli* BL21 (DE3) cells, and 5 μg of each purified protein were loaded on (A) SDS–PAGE or (B) native SDS–PAGE gels for analysis.

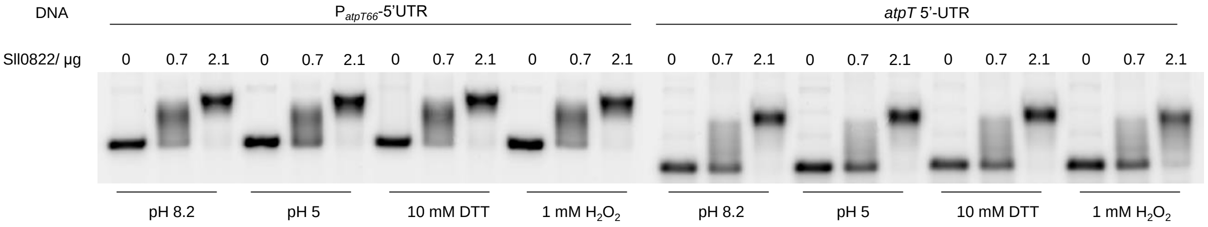

FIG S5 Electrophoretic mobility shift assay. The binding of cyAbrB2 to the P_{atpT66}-5'UTR or *atpT* 5'UTR DNA fragments under different conditions was tested by performing gel shift assays.

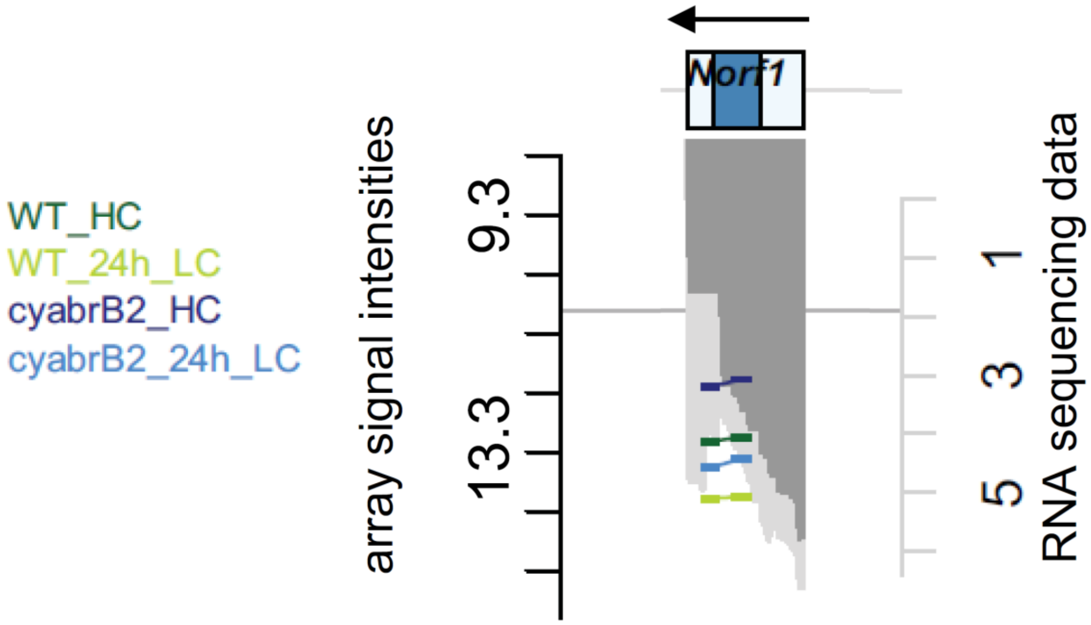

FIG S6 Genomic locus of *atpT* in Orf *et al.* (4)(called *norf1* there) with mapped array probes (note that the gene is located on the reverse strand, the direction of transcription is indicated by the horizontal arrow). *Synechocystis* 6803 WT and the Δ*cyabrB2* mutant were compared in the presence of high (HC, 5% CO_2) and low (LC, 24 h ambient air) inorganic carbon supplies. The array signal intensities are presented as $\log_2$ values and color-coded as indicated. The gray graphs represent RNA sequencing data presented as $\log_2$ read numbers, which were extracted from Mitschke *et al.* (5).

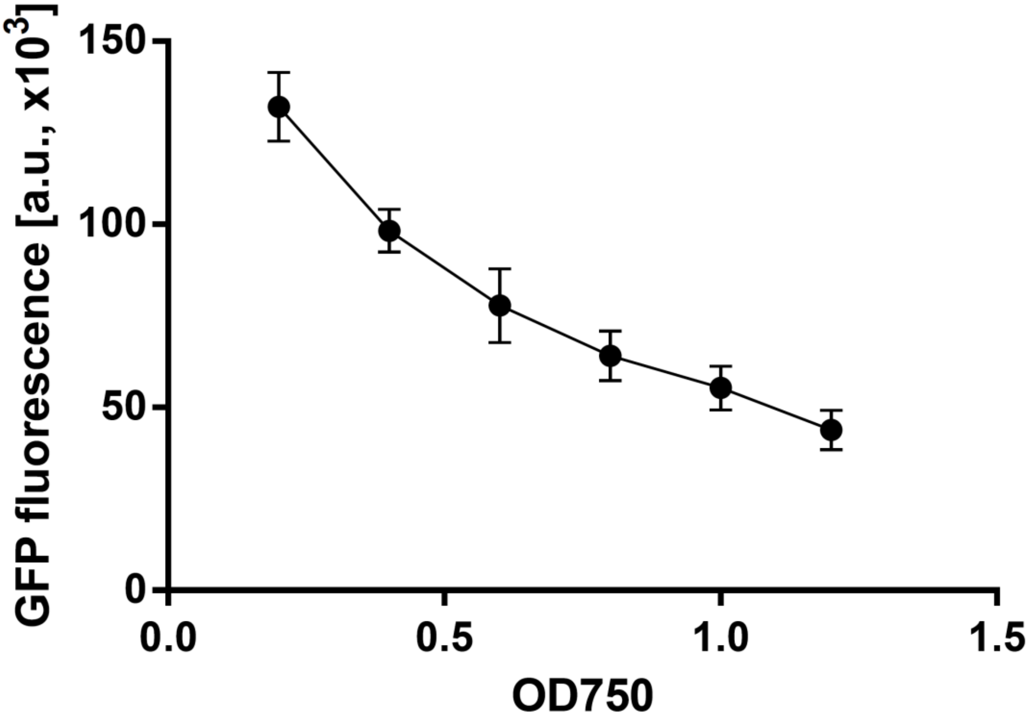

FIG S7 The correlation between GFP intensity and OD750. The exponential phase culture of the strain containing GFPconstruct_1 (OD750 approximately 1.2) was diluted as indicated, and the GFP intensities were measured immediately afterward.

References in this supplement

1. Kopf M, Klähn S, Scholz I, Hess WR, Voß B. 2015. Variations in the non-coding transcriptome as a driver of inter-strain divergence and physiological adaptation in bacteria. Sci Rep 5:9560.

2. Kopf M, Klähn S, Scholz I, Matthiessen JKF, Hess WR, Voß B. 2014. Comparative analysis of the primary transcriptome of *Synechocystis* sp. PCC 6803. DNA Res 21:527–539.

3. Edgar RC. 2004. MUSCLE: multiple sequence alignment with high accuracy and high throughput. Nucleic Acids Res 32:1792–1797.

4. Orf I, Schwarz D, Kaplan A, Kopka J, Hess WR, Hagemann M, Klähn S. 2016. CyAbrB2 contributes to the transcriptional regulation of low CO_2 acclimation in *Synechocystis* sp. PCC 6803. Plant Cell Physiol 57:2232–2243.

5. Mitschke J, Georg J, Scholz I, Sharma CM, Dienst D, Bantscheff J, Voß B, Steglich C, Wilde A, Vogel J, Hess WR. 2011. An experimentally anchored map of transcriptional start sites in the model cyanobacterium *Synechocystis* sp. PCC6803. Proc Natl Acad Sci USA 108:2124–2129.
